# A Machine Learning Method for the Detection of Brown Core in the Chinese Pear Variety Huangguan Using a MOS-Based E-Nose

**DOI:** 10.3390/s20164499

**Published:** 2020-08-12

**Authors:** Hao Wei, Yu Gu

**Affiliations:** 1 College of Information Science and Technology, Beijing University of Chemical Technology, Beijing 100029, China; weihao1@mail.buct.edu.cn; 2 Beijing Advanced Innovation Center for Soft Matter Science and Engineering, Beijing University of Chemical Technology, Beijing 100029, China; 3 Department of Chemistry, Institute of Inorganic and Analytical Chemistry, Goethe-University, Max-von-Laue-Str. 9, 60438 Frankfurt, Germany

**Keywords:** electronic nose, pear brown core, machine learning, neural network, principal component analysis

## Abstract

The brown core is an internal disorder that significantly affects the palatability and economic value of Chinese pears. In this study, a framework that includes a back-propagation neural network (BPNN) and extreme learning machine (ELM) (BP-ELMNN) was proposed for the detection of brown core in the Chinese pear variety Huangguan. The odor data of pear were collected using a metal oxide semiconductor (MOS) electronic nose (E-nose). Principal component analysis was used to analyze the complexity of the odor emitted by pears with brown cores. The performances of several machine learning algorithms, i.e., radial basis function neural network (RBFNN), BPNN, and ELM, were compared with that of the BP-ELMNN. The experimental results showed that the proposed framework provided the best results for the test samples, with an accuracy of 0.9683, a macro-precision of 0.9688, a macro-recall of 0.9683, and a macro-F1 score of 0.9685. The results demonstrate that the use of machine learning algorithms for the analysis of E-nose data is a feasible and non-destructive method to detect brown core in pears.

## 1. Introduction

Pears are the second most consumed pome fruit worldwide [1], with consumption by the adult population ranging from 23 to 108 g/day [2]. Pears have many beneficial properties for human health, such as antitussive, anti-inflammatory, antihyperglycemic, and diuretic [3]. As an ancient fruit in temperate regions [4], there are many groups of pears, such as the white pear (Pyrus bretschneideri Rehd.), sand pear (Pyrus pyrifolia (Burm. f.) Nakai), and Ussurian pear (Pyrus ussuriensis Maxim.). The Chinese pear variety Huangguan (Pyrus bretschneideri Rehd.), which is a hybrid between Pyrus bretschneideri Rehd. and Pyrus pyrifolia (Burm. f.) Nakai [5], is widely planted in northern China [6] and is popular among consumers because of its crispy and juicy flesh, good taste, and salubrity [7,8]. However, brown core, which is a physiological disease caused by high concentrations of CO_2_ [9] and enzymes [10], occurs during the transportation and storage of pears, significantly affecting the palatability and economic value of the fruit [11]. It is difficult to detect browning in pears based on their appearance, and destructive methods are required because browning of pears usually spreads from the core to the flesh [12]. Therefore, it is of practical significance to develop a non-destructive method to detect brown core in the Chinese pear Huangguan.

An electronic nose (E-nose), designed to mimic human olfactory perception and identify volatile gases, is a device which mainly contains three parts: a sampling system, a sensors array unit and a data acquisition and processing system [13]. The principle of E-nose is that the sensor array detects an odor composed of numerous different volatiles in a sample’s headspace and provides a “fingerprint” which is employed to mine potential information about samples based on appropriate algorithm [14]. E-nose devices play an important role in fruit quality detection due to their advantages of non-destructive analysis [15] and portability [16]. Voss et al. [17] obtained odor data from peaches using a metal oxide semiconductor (MOS) E-nose and designed a model to monitor the growth cycle of peaches using the Random Forest method, which achieved an accuracy of 98.08% on sample test step. Feng et al. [18] proposed a method for evaluating the freshness of cherry tomatoes using a MOS-based E-nose and analyzing the data with an extreme learning machine (ELM) and the partial least-squares method, which proved that the combination of the E-nose and the ELM was reliable for evaluating the quality and freshness of cherry tomatoes during the cold storage. Chen et al. [19] demonstrated that using an E-nose and headspace solid-phase micro-extraction combined with gas chromatography-mass spectrometry (HS-SPME/GC-MS) was a fast and accurate method to examine the volatilization of ten varieties of fresh jujube. To the best of our knowledge, most previous studies that utilized E-nose devices have focused on the identification of fruit ripeness, fruit freshness, and fruit flavor; however, few studies investigated the detection of brown core in the Chinese pear variety Huangguan using an E-nose.

Machine learning (ML) is an application of artificial intelligence (AI) and data science [20]; it is an interdisciplinary method that includes probability theory, statistics, approximation theory, and many other disciplines [21]. ML has become one of the fastest-growing technical fields [20]. An artificial neural network (ANN) is considered a flexible mathematical tool that is based on the neurons in the brain and has been used to analyze data in numerous fields [22]. ANNs can describe complex relationships between inputs and outputs. ANNs provide better performance than traditional ML methods and have been widely applied in many fields, such as food science [23], medicine [24], and chemistry [25].

In this study, we combine a back-propagation neural network (BPNN) with an ELM and propose the BP-ELMNN framework to detect the browning of Chinese pears (Huangguan) using a MOS-based E-nose. In the framework, a shallow BPNN is designed to provide sufficient pear odor information for the ELM and improve the classification performance of the ELM. The adaptive moment estimation (Adam) algorithm [26], an efficient stochastic optimization algorithm, is used for weight training instead of the traditional gradient descent method. We use the synthetic minority oversampling technique (SMOTE) [27] to increase the number of minority class samples and avoid unbalanced samples. The performances of three other neural networks (BP, ELM, and radial basis function neural network (RBFNN)) are compared with that of the proposed BP-ELMNN framework.

## 2. Materials and Methods

### 2.1. Materials and Preparation

We used 342 Chinese pears (Huangguan variety) (referred to as Chinese pears or pears hereafter) (harvested in Zhao county, Hebei province, China) with similar color and size and without insect pests and mechanical damage. The pears were stored (temperature: 0 °C, relative humidity: 90%) prior to the experiment. We used 240 pears as training samples (after 239 days of storage) and 102 pears as test samples (after 251 days of storage).

As shown in Table 1, the browning levels of the Chinese pears were determined by experts at the National Pear Improvement Center at the Hebei Academy of Agricultural and Forestry Sciences. Class 0 represents healthy pears, and class 1 to class 5 are five classes representing increasing degrees of browning. * denotes whether the browning can be discerned by the appearance of the pears. The number of samples for each class are described in Section 4.

### 2.2. E-nose Analysis and Data Acquisition

A PEN3 E-nose (Airsense Analytics GmbH, Schwerin, Germany [28]) was used to collect the odor information of the pears. An array of 10 different MOS sensors is the critical component of the PEN3 E-nose; the details of the 10 sensors are shown in Table 2 [28].

**Figure 1:**
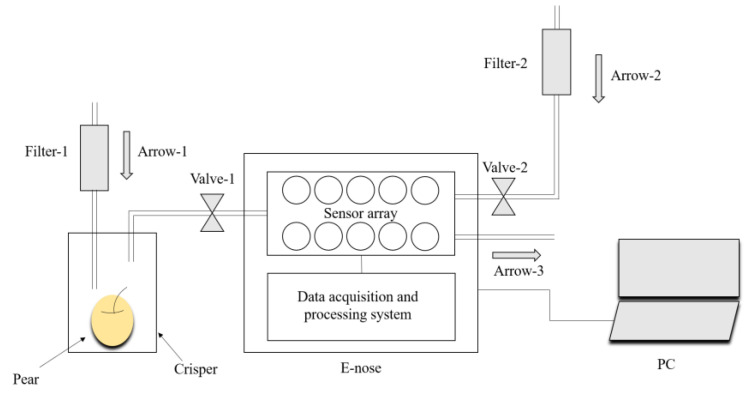
Schematic diagram of the sample collection using E-nose.

**Figure 2:**
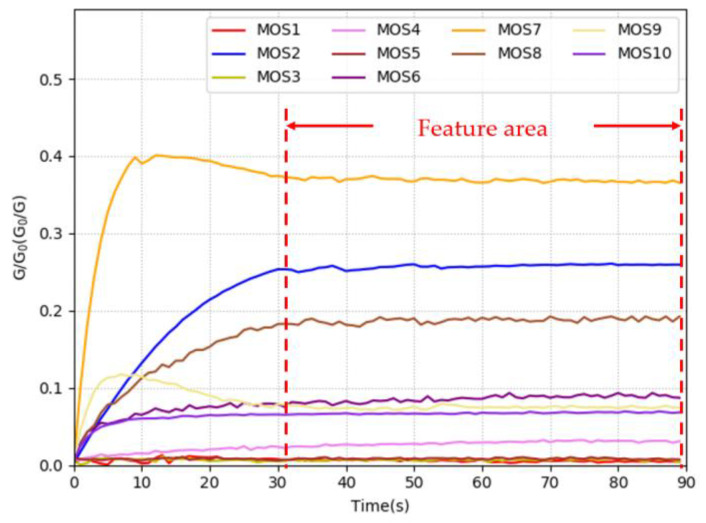
An example of the response curves of the E-nose for pears during 90 s of collecting stage.

All the experiments were conducted in the author’s laboratory (temperature: 25 ± 1 °C, relative humidity: 50 ± 2%). Figure 1 presents the schematic diagram of the collection of odor information using the E-nose. Before using the E-nose to collect data, the pear was placed into a crisper (polypropylene, 150 mm × 150 mm × 180 mm) for 900 s to allow the volatile odor of the pears to fill the crisper. We used the 15 polypropylene crispers to place the sample pears in our experiments. The crisper was cleaned with air filtered by activated carbon after a pear was sampled and not be reused until the remaining 14 crispers were used. The E-nose operation consisted of the collection and cleaning stages. During the collection stage, air flows into the crisper in the direction of arrow-1 and flows out of the E-nose in the direction of arrow-3. During this stage, valve-1 is in the open state and valve-2 is in the closed state. The sample’s volatile gas was drawn into the E-nose at a constant flow rate of 10 mL/s and came into contact with the ten MOS sensors in the chamber. As the sensor surface became saturated, the conductivity increased, and the value stabilized. The collection stage lasted 90 s. During the cleaning stage, air filtered by activated carbon was drawn into the E-nose in the direction of arrow-2 to remove the substances adsorbed on the surface of the sensor. During this stage, valve-2 is in the open state and valve-1 is in the closed state. The cleaning stage also lasted 90 s to ensure that the material adsorbed on the surface of the sensor was removed entirely. The cleaning stage occurred after the completion of the collection stage. According to the characteristics of the MOS sensor, the response curve of the sensor increases rapidly initially and then flattens gradually in the collection stage. The stable value (SV) is a crucial and simpler feature parameter of the E-nose response signal, which can reflect the properties of substances in the volatile gas and be used in pattern recognition algorithms appropriately. We used the SV of the response signal as the input data of the neural network. Since the response signal of the PEN3 E-nose entered the stable state at 30 s, we used the data from 31 s to 90 s as the SVs. An example of the response curves of the E-nose for pears during 90 s of collecting stage was showed in Figure 2. Each curve represents the ratio of G (the conductivity of sensors contact with sample’s volatile gas) to G_0_ (the conductivity of sensors contact with air filtered by activated carbon) and the feature area was displayed between the two red dotted lines. Therefore, the data we obtained were expressed as a 14,400 × 10 matrix for training, and a 6120 × 10 matrix for testing (the sampling period of the E-nose was 1 s).

**Figure 3:**
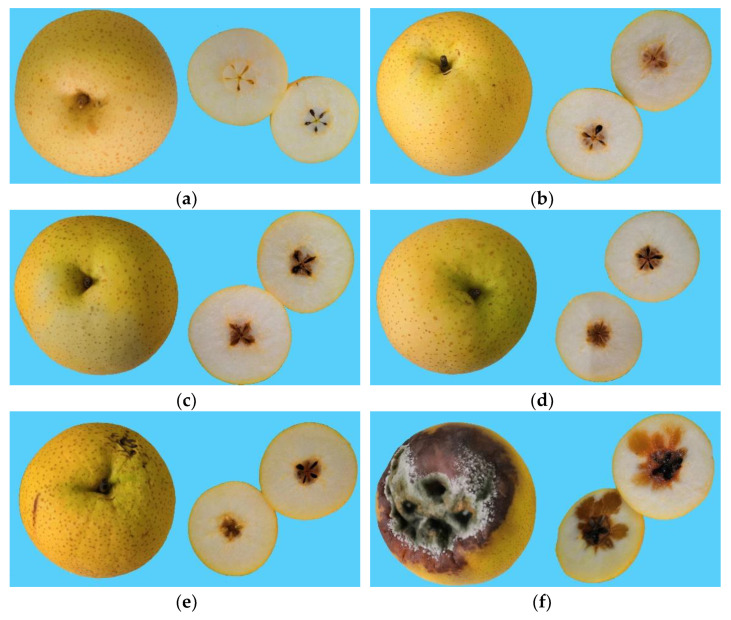
Photos of the Chinese pear (Huangguan) with different browning levels. Photos (**a**–**f**) indicates the levels of pear browning from class 0 to class 5, respectively.

After collecting the data using the E-nose, the pears were immediately cut open and labeled by experts (the label result were showed in Table 3). Photos of the Chinese pears in the six classes of browning levels were obtained and shown in Figure 3b–d. It is challenging to determine to brown of the core based on the appearance in class 1 to class 3.

### 2.3. Adaptive Moment Estimation Algorithm

The selection of an efficient and suitable optimization algorithm is crucial for neural network training. The Adam algorithm combines the advantages of the AdaGrad method [29] and the RMSProp method [30] and is an optimization algorithm that only requires first-order gradients with little memory requirement [26]. The gradient is not used to update the parameters for the optimization of the objective function in the Adam algorithm. The moment estimation, which is defined in Equations (1) and (2), is used to update the parameters:

(1) $$m_{t}=\varepsilon_{1}m_{t-1}+\left(1-\varepsilon_{1}\right)g_{t}$$

(2) $$v_{t}=\varepsilon_{2}v_{t-1}+\left(1-\varepsilon_{2}\right)g_{t}^{2}$$

where *t* and $g_{t}$ denote the timestep and gradient, respectively. m and v are the first-order moment estimate and second-order moment estimate, respectively. $\varepsilon_{1}$ and $\varepsilon_{2}$ are attenuation coefficients. In the initial phase, $m_{t}$ and $v_{t}$ tend to be biased towards the starting values [31]. Therefore, the moment estimation is modified as follows:

(3) $${\hat{m}}_{t}=\frac{m_{t}}{1-\varepsilon_{1}^{t}}$$

(4) $${\hat{v}}_{t}=\frac{v_{t}}{1-\varepsilon_{2}^{t}}$$

The parameter update rule is defined as:

(5) $$\vartheta_{t}=\vartheta_{t-1}-\frac{\sigma {\hat{m}}_{t}}{\sqrt{{\hat{v}}_{t}}+\epsilon }$$

where σ is the learning rate. As an extension of the stochastic gradient descent method [32], the Adam algorithm possesses fast speed in the optimization process without falling into the local optimum [33].

### 2.4. Synthetic Minority Oversampling Technique

Unbalanced sample data adversely affect the training performance in neural networks. This problem can be solved by the oversampling technology to supplement minority samples. The SMOTE is one of the most widely used oversampling methods [34] and generates a new sample $x_{i}$ randomly between a minority class sample $x_{m}$ and a k-nearest neighbor minority class sample $x_{n}$ of $x_{m}$ as follows:

(6) $$x_{i}=x_{m}+\alpha \times \left(x_{m}-x_{n}\right)$$

where α is a random number that is greater than 0 and smaller than 1. After oversampling, the features of each class of data can be learned by the neural networks.

### 2.5. Back-Propagation Neural Network

The BPNN is one of the most mature and well-known feed-forward neural networks [35]; it is wildly used for prediction and classification [36,37]. A BPNN consists of an input layer, a hidden layer, and an output layer, and all layers are connected. The data in the input layer of the BPNN are processed layer by layer using forward propagation until the output layer is obtained. The data features are extracted using a layer by layer calculation process [38]. The back-propagation algorithm in the BPNN is used to calculate the weights required in the network according to the error between the network output and expected output.

### 2.6. Extreme Learning Machine

An ELM is a single hidden layer neural network, which has better training performance than traditional algorithms [17,18,39]. One of the most significant features of the ELM is that the weights do not need to be obtained by iterative updating. The optimal solution of the ELM is obtained by one-time learning [40]. The input weights and deviations of the hidden layer of the ELM are generated randomly, and the input weights of the output layer are obtained analytically [40]. Due to the powerful performance of the ELM, it has been widely used in many fields [22].

### 2.7. Radial Basis Function Neural Network

The RBFNN is a three-layer feed-forward neural network. The input layer of the RBFNN does not process the data but maps the data directly to the hidden layer [41]. Each neuron in the hidden layer represents a radial basis function, and the data is sent to the output layer after the calculation of the radial basis function. A simple linear transformation of the hidden layer output is performed to obtain the output layer [42]. The center and width of the hidden layer neurons in the RBFNN have a significant influence on the training results of the network [43,44].

## 3. Proposed Method

It is well known that in traditional BPNNs, a large number of hidden layers result in excess features learned by the network, leading to overfitting of the network. However, if there are too few hidden layers, the performance of the BPNN could be affected. The ELM has better generalization performance than the BPNN [22], but the structural characteristics of a single hidden layer may result in an inability of the network to extract sufficient features of the data.

**Figure 4:**
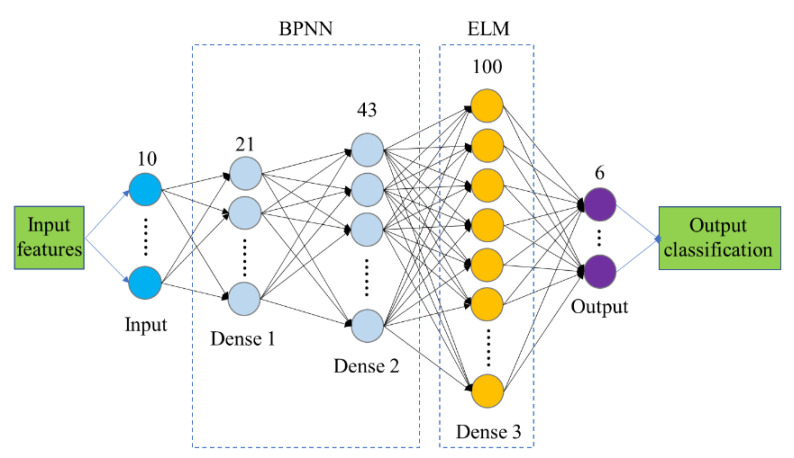
The proposed BP-ELMNN structure for the discrimination of the categories of pears.

We combined the advantages of the two networks and developed the BP-ELMNN framework (Figure 4) to detect the degree of pear browning. The output of the hidden layer of the BPNN is input into the ELM. This framework contains three dense layers. The activation function of the neurons in the Dense 1 layer and Dense 2 layer is the Relu function, and the neurons in the Dense 3 layer are activated by a Sigmoid function. The Adam algorithm is used to iteratively update the weights and thresholds from the input layer to the Dense 2 layer. The weights and thresholds between the Dense 2 layer and Dense 3 layer are generated randomly and are not iteratively updated during network training. The input weights of the output layer are obtained using Equation (7).

(7) $$\beta =O_{dense3}^{-1}T$$

where β is the input weight of the output layer, $O_{dense3}$ denotes the output of the Dense 3 layer, and T is the label of the training data.

The loss function is the cross-entropy function, which is defined as:

(8) $$Loss_{1}=-\overset{n}{\underset{i=1}{\sum }}l_{i}\log\left(y_{i}\right)$$

where n is the number of classes, $l_{i}$ is the label of the data, and $y_{i}$ denotes the actual output of the neural network. The L2 regularization term of the weight vectors was used in the loss function to avoid overfitting:

(9) $$Loss=Loss_{1}+\alpha_{r}{\left\|W\right\|}_{2}^{2}$$

where $\alpha_{r}$ is the regularization coefficient, W denotes the weight vectors, and ${\left\|\cdot \right\|}_{2}$ is the L2 norm.

The proposed BP-ELMNN Algorithm 1 is summarized as follows:
**Algorithm 1.** BP-ELMNN**Input:** training data.**Output:** predicted category.**Begin**Step 1: train the BPNN part on the training data using the Adam algorithm.Step 2: randomly select the input weights and thresholds of the Dense 3 layer.Step 3: calculate the input weight β of the output layer using Equation (7).Step 4: test the BP-ELMNN model on the test data.Step 5: output the classification results.**End**

## 4. Result and Discussion

### 4.1. Data Analysis and Sample Supplementation

The training samples and test samples of the Chinese pears were divided into six categories according to the browning levels (see Table 1); the number of samples is shown in Table 3. Since it was not possible to obtain the same number of pears in each category, we supplemented the sample data of the minority class using the SMOTE. In the training data (TR), the number of all minority class samples was increased to obtain 4620 samples. In the test data (TE), the number was increased to 2280. Table 4 shows the changes that were made to the TR and TE using the SMOTE. The new dataset was expressed as a 27,720 × 10 matrix for the new data of TR (NTR) and a 13,680 × 10 matrix for the new data of TE (NTE).

### 4.2. Principal Component Analysis

**Figure 5:**
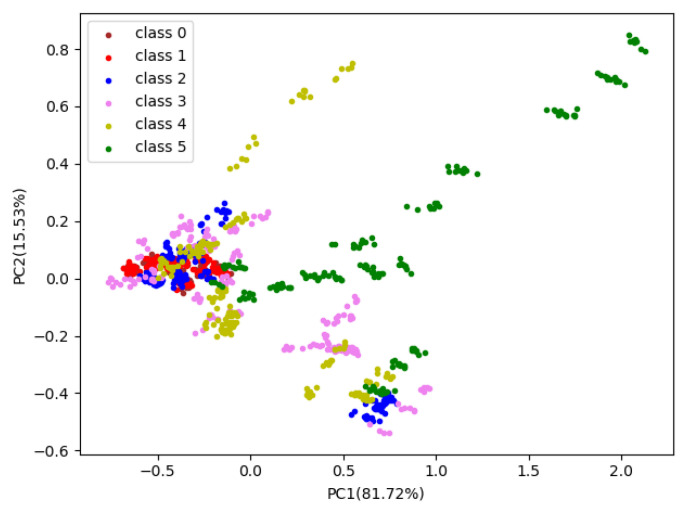
Projections of the two first primary components of the PCA computed on training set to determine the levels of pear browning. The horizontal axis represents the principal component 1 with 81.72% contribution rate and the vertical axis represents the principal component 2 with 15.53%.

Principal component analysis (PCA) is a standard feature extraction method used in pattern recognition [45] that minimizes the correlation between the components by rotating the covariance matrix [46]. We used PCA to extract the principal components of the training samples. The scatter diagram of the PCA result was showed in Figure 4, in which the dimensions were reduced from ten variables to two principal components. The contribution of the principal component 1 (PC1) was 81.72%, and that of principal component 2 (PC2) was 15.53%. In general, the first few principal components whose cumulative contribution exceeds 95% are considered to contain nearly all the information of the original data [45]. Therefore, we believe that PC1 and PC2 are representative of the characteristics of the original data. We selected randomly 18 pears (3 × 6, three pears per category from the training set) to present the PCA results as an example in Figure 5. As shown in Figure 5, the odor information of the six categories of pears has significant overlap, and the categories cannot be visually distinguished in the two-dimensional projection space based on PCA. Accordingly, we concluded that the odor of pears with different levels of browning was rich and strong. Even if the cumulative contribution rate of PC1 and PC2 reaches 97.25%, it was still not easy to distinguish pears with different levels of browning in a low-dimensional space.

**Figure 6:**
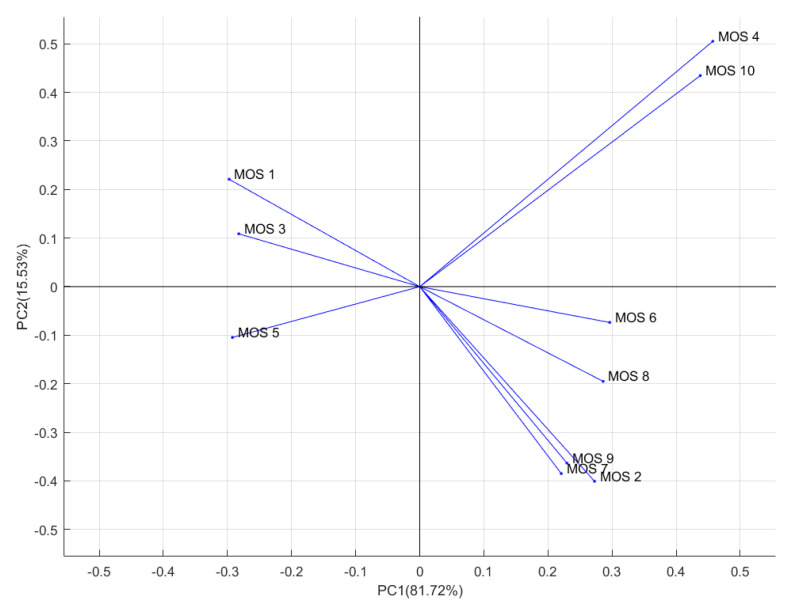
PCA loading plot of measurements about different levels of pears browning.

The PCA loading plot was shown in Figure 6. Ten vectors represented ten sensors (MOS 1, …, MOS 10) of the PEN3 E-nose. The contribution of sensors is denoted through the direction and length of vector. As shown in Figure 6, MOS 4 has the largest positive coefficient for first principal component so that MOS 4 has the largest contribution to the first principal component during the process of PCA.

### 4.3. Comparison of the Classification Results of the Four Methods

In this study, the performances of the BPNN and ELM used in the BP-ELMNN were compared with that of the proposed BP-ELMNN model. In addition, the performance of the RBFNN, which has been widely used in many applications [47], was also analyzed. The methods were implemented using a PC (Intel Core i7-8550U processor), and Python and Tensorflow were used for programming.

For the BPNN model, a network structure with three hidden layers (21, 43, and 87 neurons in the hidden layers) was designed to detect the degree of browning of the pears. The activation function of the neurons in the hidden layers was the Relu function, and the neurons in the output layer were activated by the Softmax function. The BPNN model was trained and validated using 10-fold cross-validation on the NTR and achieved a training accuracy of 0.7845. The experimental results of the NTE are shown in Table 5. An accuracy of 0.7623, a macro-precision of 0.6732, a macro-recall of 0.7623, and a macro-F1 score of 0.7150 on NTE were obtained. The BPNN model provided a medium classification accuracy for detecting brown core in the Chinese pears.

The ELM model had 500 neurons in the single hidden layer and was activated by a Sigmoid function. The input weights of the hidden layer and the output layer were obtained randomly and analytically, respectively. As shown in Table 5, the ELM model on the NTE provided the second-best classification result.

**Figure 7:**
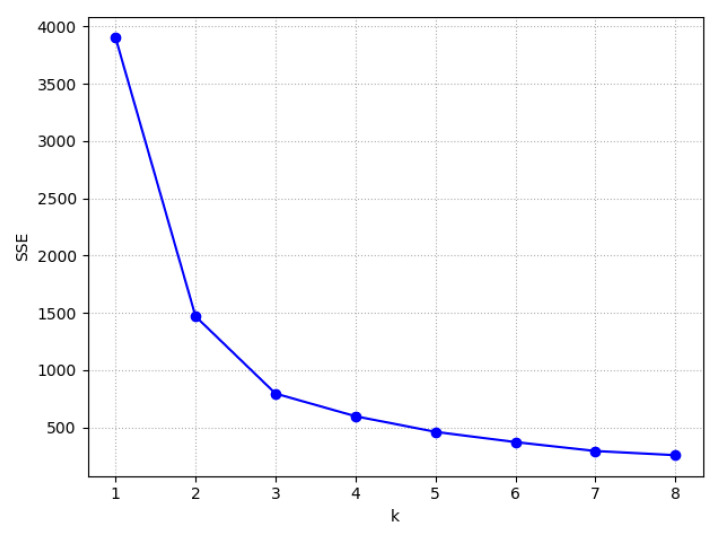
The relation curve between the SSE and k.

In the RBFNN model, a Gaussian function was used as the activation function of the hidden layer neurons. The center of the activation function was obtained via the k-means algorithm. The sum of squared errors (SSE) was calculated as follows:

(10) $$SSE=\sum_{i=1}^{k}\sum_{d\in Q_{i}}{\left(d-c_{i}\right)}^{2}$$

where $Q_{i}$ is the ith cluster, d is a sample of $Q_{i}$, $c_{i}$ is the mean of all samples of $Q_{i}$, *k* is the number of cluster center. Figure 7 shows the relation curve between the SSE and k; a value of *k* = 3 was used because the rate of decrease of the SSE was relatively low when *k* > 3. The width of the activation function was obtained using Equation (11):

(11) $$width_{k}=\frac{\mu_{max}}{\sqrt{k}}$$

where $\mu_{max}$ is the maximum distance between the centers. The Softmax function was utilized to activate the output layer neurons. The classification results of the RBFNN for the NTE is shown in Table 5; this algorithm provided the lowest classification accuracy.

**Figure 8:**
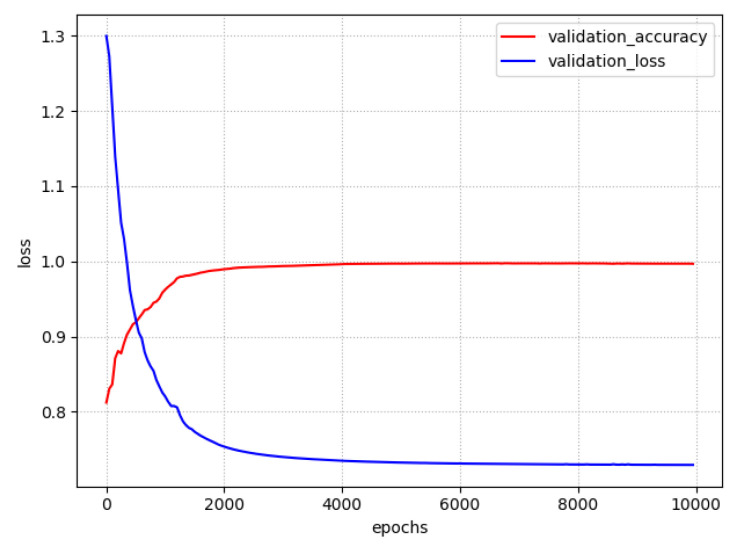
The loss and cross validation curves of the BP-ELMNN during training process.

For training the BP-ELMNN, 10-fold cross-validation was used to evaluate the performance of the network on NTR. The curves of validation loss and accuracy during training process are shown in Figure 8. After 8000 epochs, the validation loss did not decrease further, and the accuracy for the NTR was 0.9874, indicating convergence. As shown in Table 5, the BP-ELMNN achieved the highest classification accuracy for the NTE, with an accuracy of 0.9683, macro-precision of 0.9688, macro-recall of 0.9683, and a macro-F1 score of 0.9685.

The experimental results of the four methods are summarized in Table 5; we used four evaluation indicators (accuracy, macro-Precision, macro-recall, and macro-F1 score) to assess the classification performance of the models. The accuracy represents the ratio of the correctly classified samples to the total samples [48]. The precision is the ratio of the true positive results to all positive results [49], and the mean value of the precision of each sample label is the macro-precision. The recall represents the number of positive results that were correctly classified as positive in the experimental result [48], and the mean value of the recall of each sample label is the macro-recall. The macro-F1 score is the harmonic mean of the macro-precision and macro-recall.

**Figure 9:**
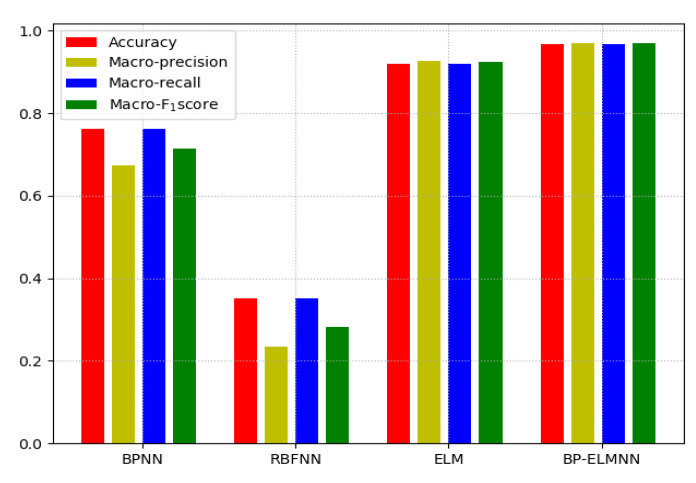
Bar chart of the performance of the four methods (accuracy, macro-precision, macro-recall, and macro-F1 score) for the NTE.

The performance of the four neural networks is shown in Figure 9 in a bar chart. The results indicate that the BP-ELMNN had a strong nonlinear fitting ability for the detection of brown core in the Chinese pears.

We also used the four neural networks to classify the data not processed by the SMOTE (the TR and TE) to determine the performance of the BP-ELMNN framework when unbalanced data are used. The classification results for the original data of the TE (OTE) are shown in Table 6. Unbalanced data often results in an inability of the neural network to learn the information of the different types of data and lower classification accuracy. However, Table 6 indicates that the proposed BP-ELMNN framework also provides the best performance for the unbalanced data, with an accuracy of 0.9285, macro-precision of 0.9188, macro-recall of 0.9017, and a macro-F1 score of 0.9089.

## 5. Conclusions

In this paper, we proposed a multi-layer BP-ELMNN framework to detect brown core in Chinese pears (Huangguan) using a MOS-based E-nose device. PCA was used to extract the principal components, whose cumulative contribution was higher than 95%. However, pears with different degrees of browning could not be distinguished by the PCA. The result of PCA showed that the odors of pears with different degrees of browning were indistinguishable in two-dimensional space (Even if their cumulative contribution rate reached 97.25%) and highly similar. The proposed BP-ELMNN framework obtained the highest classification accuracy among the four neural networks, indicating that the hidden layer structure of the BPNN extracted sufficient data, which were used as input for the ELM. The original data that were not processed by the SMOTE were used in a comparative experiment to demonstrate the robustness of the BP-ELMNN framework to unbalanced data.This study demonstrated that the use of the E-nose and a suitable ANN algorithm provided excellent performance for the non-destructive detection of brown core in the Huangguan Chinese pear. The findings provide insights into the use of ANNs in the food industry, helps manufacturers monitor pear quality, and promotes the practical application of E-nose devices.

## Figures and Tables

**Table 1:** Browning levels of the Chinese pear Huangguan.

Class	Standard			*
	Appearance	Core	Flesh	
0	Good	Good	Good	-
1	Good	Light brown	Good	No
2	Good	Brown	Good	No
3	Good	Dark brown	Good	No
4	Brown	Dark brown	Dark brown	Yes
5	Dark brown	Dark brown	Dark brown	Yes

* denotes whether browning can be discerned by the appearance.

**Table 2:** The sensor array of the PEN3 electronic nose (E-nose).

Number	Sensor	Substance Sensitivity
MOS1	W1C	Aroma constituent
MOS2	W5S	Sensitive to nitride oxides
MOS3	W3C	Ammonia, aroma constituent
MOS4	W6S	Hydrogen
MOS5	W5C	Alkane, aroma constituent
MOS6	W1S	Sensitive to methane
MOS7	W1W	Sensitive to sulfide
MOS8	W2S	Sensitive to alcohol
MOS9	W2W	Aroma constituent, organic sulfur compounds
MOS10	W3S	Sensitive to alkane

**Table 3:** Label result and number of samples.

Training Samples.		Test Samples	
Class	Number	Class	Number
0	9	0	3
1	21	1	8
2	42	2	15
3	77	3	38
4	49	4	20
5	42	5	18

**Table 4:** The changes that were made to the training data (TR) and test data (TE).

Class	TR			TE		
	The Original Number of Samples	Added Number of Samples	New Number of Samples	The Original Number of Samples	Added Number of Samples	New Number of Samples
0	540	4080	4620	180	2100	2280
1	1260	3360	4620	480	1800	2280
2	2520	2100	4620	900	1380	2280
3	4620	0	4620	2280	0	2280
4	2940	1680	4620	1200	1080	2280
5	2520	2100	4620	1080	1200	2280

**Table 5:** The accuracy, macro-precision, macro-recall, and macro-F1 score of the four methods for the NTE.

Methods	Accuracy	Macro-Precision	Macro-Recall	Macro-F1 Score
BPNN	0.7623	0.6732	0.7623	0.7150
RBFNN	0.3504	0.2347	0.3504	0.2811
ELM	0.9190	0.9272	0.9190	0.9231
BP-ELMNN	0.9683	0.9688	0.9683	0.9685

**Table 6:** The accuracy, macro-precision, macro-recall, and macro-F1 score of the four methods for the OTE.

Methods	Accuracy	Macro-Precision	Macro-Recall	Macro-F1score
BPNN	0.7399	0.6398	0.7055	0.6711
RBFNN	0.3493	0.1255	0.2153	0.1586
ELM	0.8809	0.8653	0.8677	0.8665
BP-ELMNN	0.9285	0.9188	0.9017	0.9089
